# Reactive Nodular Fibrous Pseudotumor Presenting as Gastric Outlet Obstruction in a Young Adult Male Patient: A Case Report and Review of the Medical Literature

**DOI:** 10.1155/crip/6943449

**Published:** 2026-02-27

**Authors:** David Saulino, David Gonzalo, Michael Still, Meredith Thompson, Christopher Forsmark, Alexander Ayzengart, Michael Feely

**Affiliations:** ^1^ University of Florida, Gainesville, Florida, USA, ufl.edu; ^2^ Renown Surgery Care, Reno, Nevada, USA

## Abstract

Mesenchymal proliferations involving the gastrointestinal tract are uncommon and often unexpected upon initial clinical presentation. Here, we are reporting a case of a 20‐year‐old male patient presenting to the hospital with abdominal pain and vomiting. Upon evaluation, the patient was initially discharged home on antacid therapy. The symptoms progressed in intensity, and a subsequent CT scan was remarkable for marked gastric expansion and a prepyloric mass. The macroscopic examination of the resected lesion revealed a 5.6 cm multilobular fibrotic mass involving the gastric wall. Microscopic analysis was notable for a spindle‐cell lesion with admixed lymphoplasmacytic inflammation, patchy necrosis, and calcifications. The immunohistochemical workup was suggestive of a reactive nodular fibrous pseudotumor. This case is notable for several aspects, including the acute clinical presentation, intralesional calcifications, abundant IgG4‐positive plasma cells, and lack of CD117 immunohistochemical expression. The peculiarities of the case and a review of the medical literature will be presented.

## 1. Introduction

Mesenchymal lesions in the gastrointestinal tract are uncommon compared to their epithelial counterparts. Gastrointestinal mesenchymal tumors, in particular, are a heterogeneous group of entities, with lesions ranging from benign to malignant; therefore, correct classification is essential to guide appropriate management decisions [1]. Reactive nodular fibrous pseudotumor (RNFP) is classified as a benign lesion but may potentially be considered more accurately as a reactive entity rather than a true neoplasm [2].

Gastric outlet obstruction (GOO) is a term used to describe an impingement of the outflow of gastric contents, typically caused by a pathologic process involving the distal stomach or proximal duodenum [3, 4]. This process could be related to extrinsic compression or intrinsic obstruction from various etiologies [3–5]. Some common causes of GOO include peptic ulcer disease, pyloric stenosis, and foreign materials [3, 5]. Compression of the pylorus from neoplasms arising from pathologies of adjacent organs (such as the liver or pancreas) has also been reported [3, 5–7]. Common clinical symptoms of GOO include abdominal pain, nausea, early satiety, and vomiting [3, 5].

Here, we are reporting a case of RNFP showing intralesional calcifications and smooth muscle actin (SMA) and desmin expression in the spindle cells. Clinical and morphological features of the case will be compared to previously reported data.

## 2. Case Report

**Figure 1:**
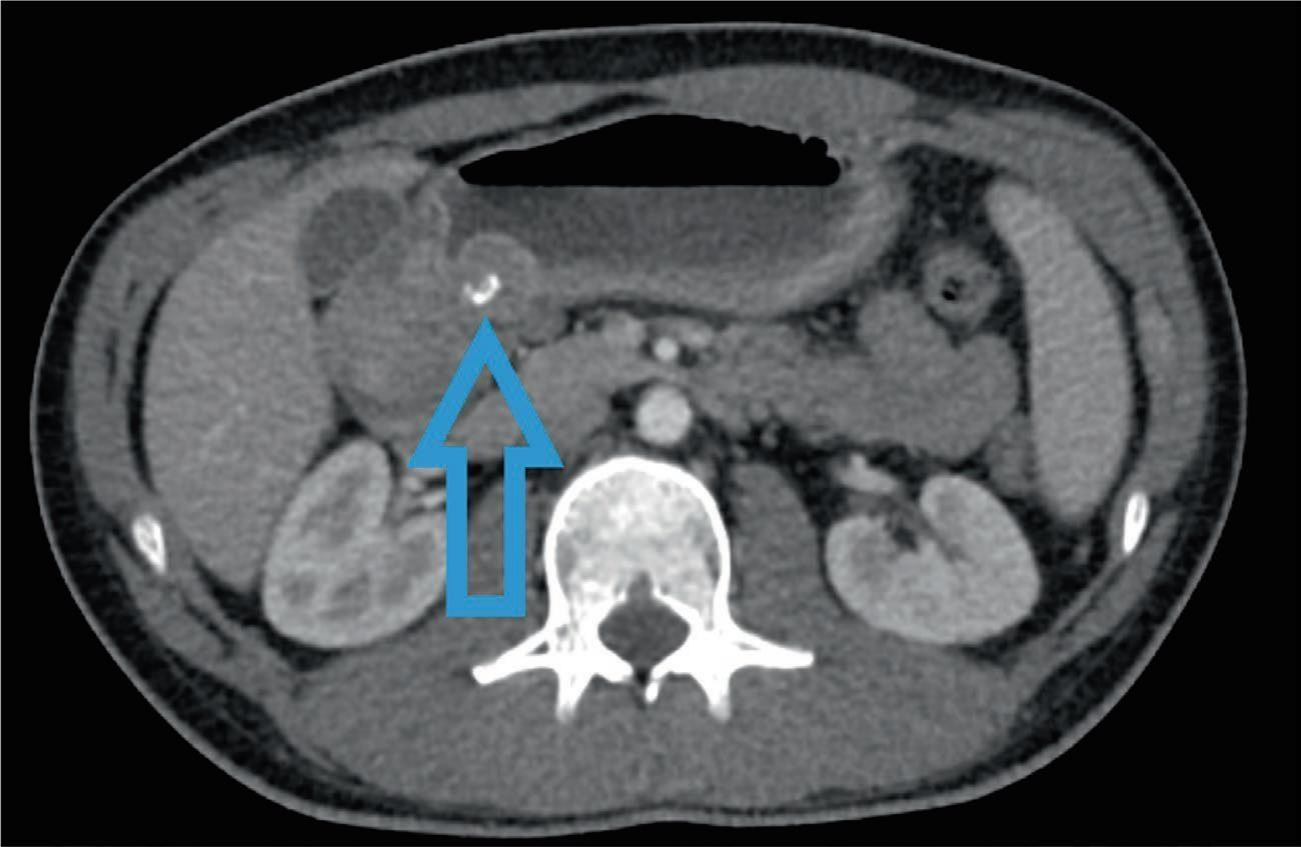
Computed tomography imaging revealed obstruction of the pyloric outlet by a 2.4 cm mass with associated calcifications (blue arrow points to calcifications).

A 20‐year‐old male patient presented to the hospital where the authors are employed with a 2‐month history of abdominal pain, belching, and vomiting. He offered a history of possible abdominal wall hernia or diastasis several years previously but had not had symptoms since. The symptoms the patient reported at initial presentation had become so severe that he started sleeping in an upright position for relief. This was also accompanied by a 20‐lb (9 kg) weight loss. In the 2 weeks prior to initially presenting to the hospital, the patient started taking a proton pump inhibitor (omeprazole), which offered mild but not substantial relief. He was subsequently discharged with advice to begin taking antacids and a nighttime H2 blocker. A follow‐up appointment a week later revealed new symptoms of visible peristalsis. A computed tomography (CT) scan of the abdomen and pelvis revealed significant gastric distension and a prepyloric mass that was considered suspect for a neoplastic process (Figure 1). The mass measured 2.4 cm on the aforementioned abdominopelvic CT scan and was heterogeneous with scattered coarse calcifications. The initial radiological differential diagnosis included mucinous adenocarcinoma, gastrointestinal stromal tumor (GIST), or lymphoma. Endoscopic examination was performed with sonography, which was notable for an ill‐defined intramural lesion in the gastric antrum. Subsequent endoscopic gastric mucosal biopsies of this mass were composed of multiple fragments of gastric tissue ranging from 0.1 to 0.7 cm placed in one block, with six tissue sections stained for histologic review; the microscopic diagnosis of the biopsy specimens was that of mild chronic inactive gastritis. Due to the severity of the symptoms, a surgical resection was decided as the best option. Surgical evaluation confirmed a large submucosal prepyloric mass, which was subsequently removed completely via distal gastrectomy. Perioperatively, there was no ascites or macroscopic lesions of the peritoneal, bowel wall, or liver surface.

**Figure 2:**
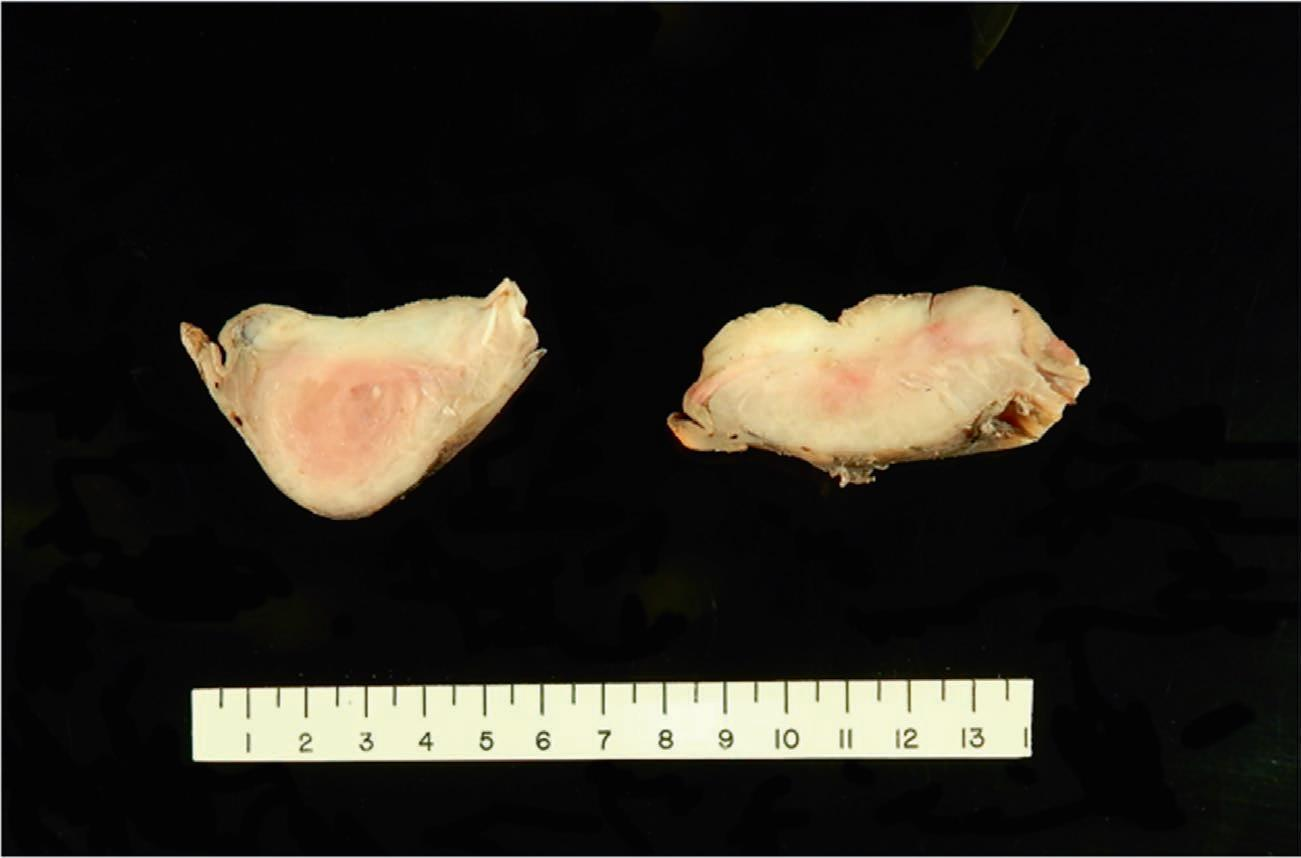
Postfixation aspect of the cut‐section of the resected specimen with a vague, ill‐defined lesion occupying the majority of the gastric wall.

**Figure 3:**
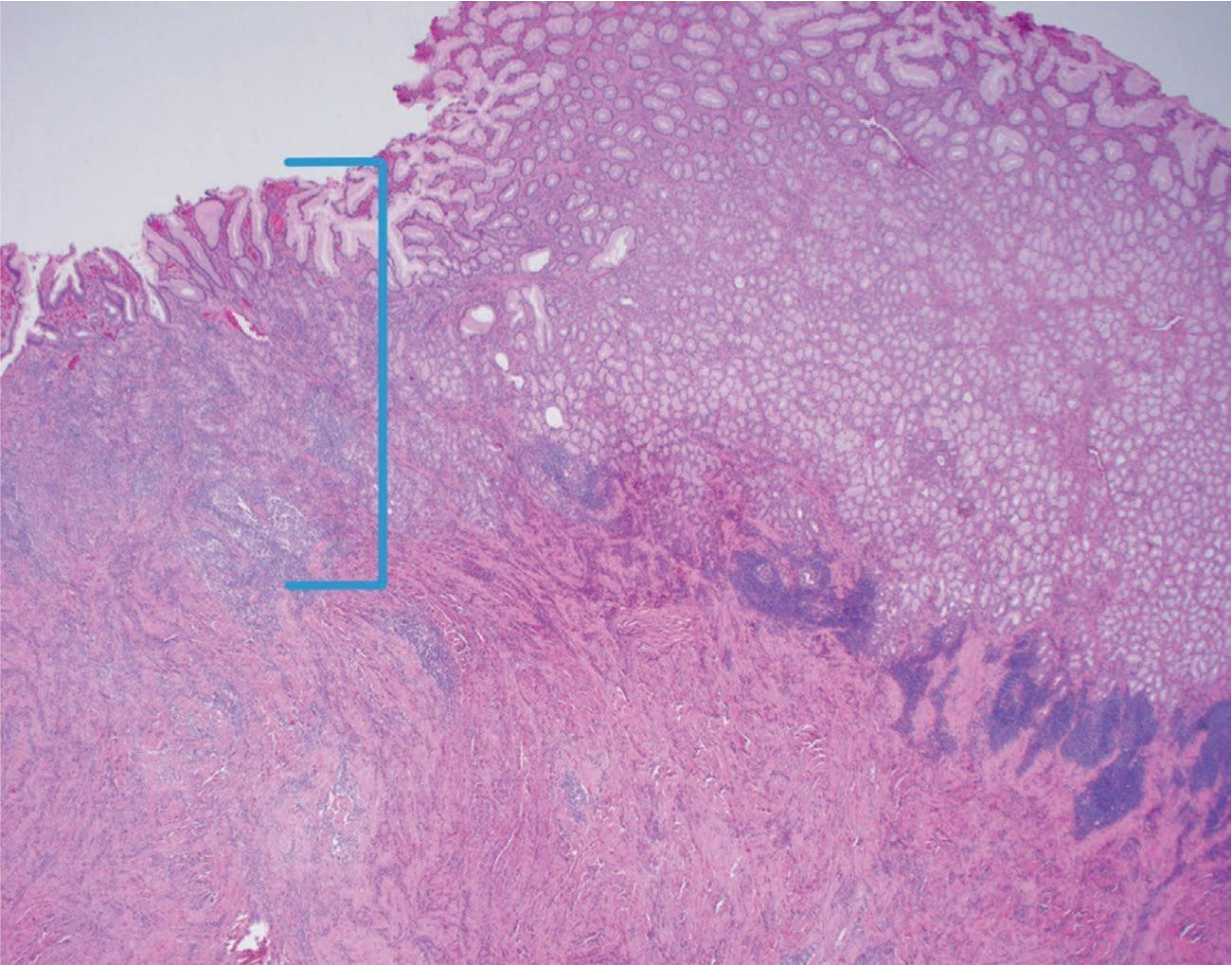
Fibroinflammatory spindle cell lesion with overlying mucosal injury (20x optical microscope magnification taken with a microscope digital camera, area of mucosal atrophy in bracket).

**Figure 4:**
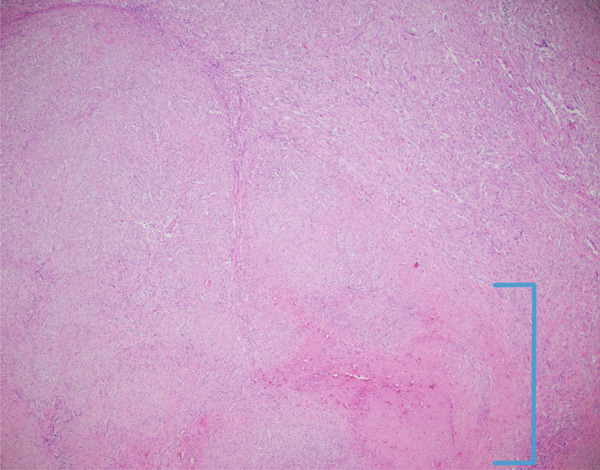
Spindle cell lesion with associated necrosis (40x magnification via a microscope digital camera, area of necrosis in bracket).

**Figure 5:**
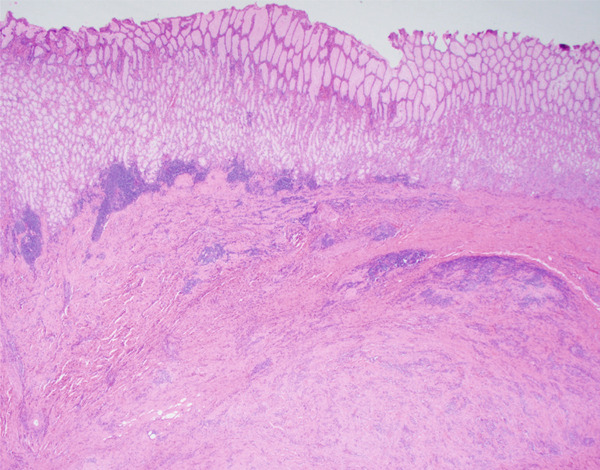
Lesion with relation to the submucosa and intact mucosal surface (20x magnification via a microscope digital camera).

**Figure 6:**
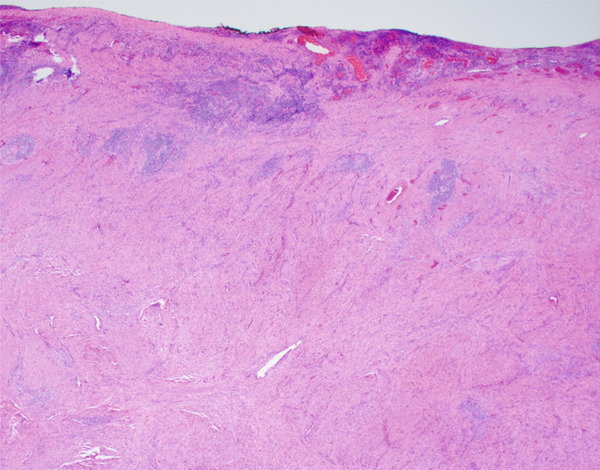
Lesion with relation to muscularis propria and serosa (20x magnification via a microscope digital camera).

**Figure 7:**
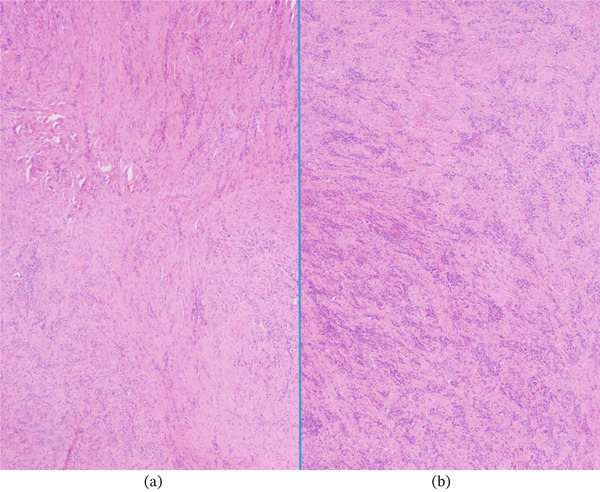
Inflammatory cells interspersed in the lesion (40x magnification via a microscope digital camera): (a) with lesser density and (b) with greater density.

**Figure 8:**
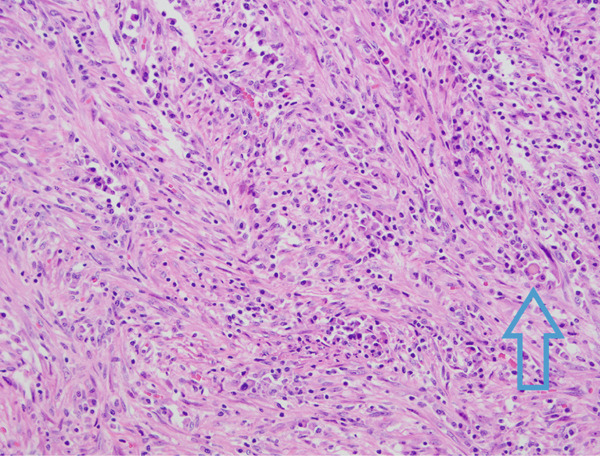
Spindle cell lesion with abundant plasma cells and occasional Russell bodies (200x magnification via a microscope digital camera, blue arrow points to Russell body).

**Figure 9:**
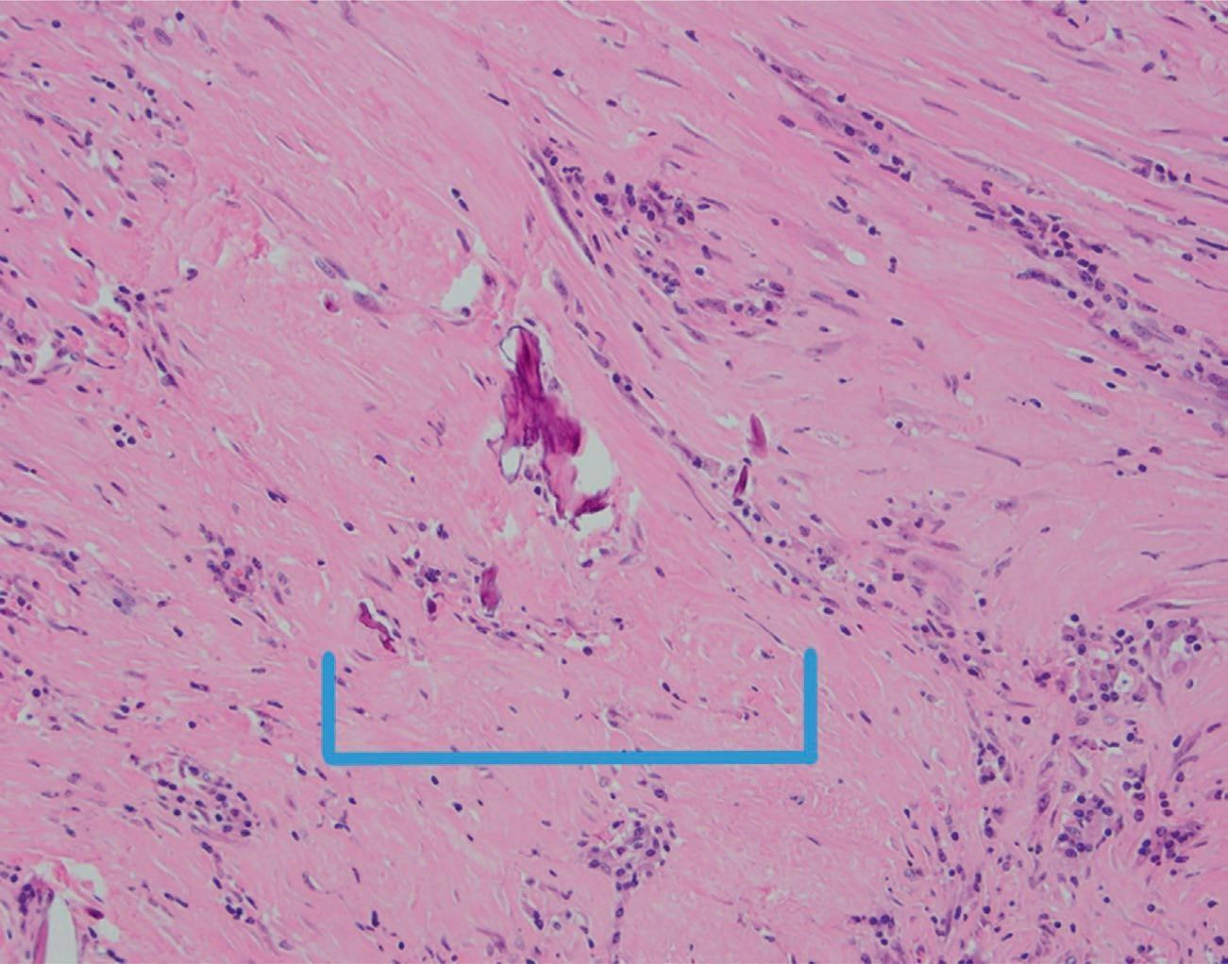
Hyalinized stroma with occasional dystrophic calcifications (100x magnification via a microscope digital camera, calcifications in bracket).

Gross examination of the specimen revealed an 8.5 cm in length × 6.1 cm in maximum diameter distal gastrectomy specimen. A 5.6 × 4.5 × 4.5 cm prepyloric multilobulated mass with indistinct borders was detected in the gastric wall. The cut surface showed a white‐pink whorled appearance with some central calcifications and a focus of hemorrhage (Figure 2). Representative sections of the tumor (eight total blocks of tumor) showed a fibrotic lesion predominantly involving the muscularis propria and submucosa. The lesion focally extended to the gastric lining, with associated chronic inflammation and compressive mucosal injury (Figure 3). The lesional spindle cells were intermixed with collagen, of generally uniform cellularity throughout the lesion, and appeared to be of myofibroblastic origin (Figure 4). The proliferation extended to the submucosa and muscularis propria on microscopic examination, and the interface with the surrounding tissue was indistinct and not well‐demarcated (Figures 5 and 6). Vessels were not prominent. The spindle cells each contained a single nucleus that displayed finely dispersed chromatin and occasional pinpoint nucleoli; no significant cellular atypia or mitotic activity was noted. The collagen bands predominantly consisted of short fibers. Abundant chronic inflammatory cells were noted between the collagen fibers, consisting largely of plasma cells with lymphocytes and rare eosinophils (Figure 7). Scattered pink spherical bodies with concretions that appeared to be intracytoplasmic were present in at least some foci, consistent with Russell bodies (Figure 8). Additionally, there were foci of coagulative necrosis with associated fibrin, hemorrhage, and myofibroblastic reaction comprising approximately 15% of the overall mass on representative sections. Scattered foci of dystrophic calcifications were also present (Figure 9).

**Figure 10:**
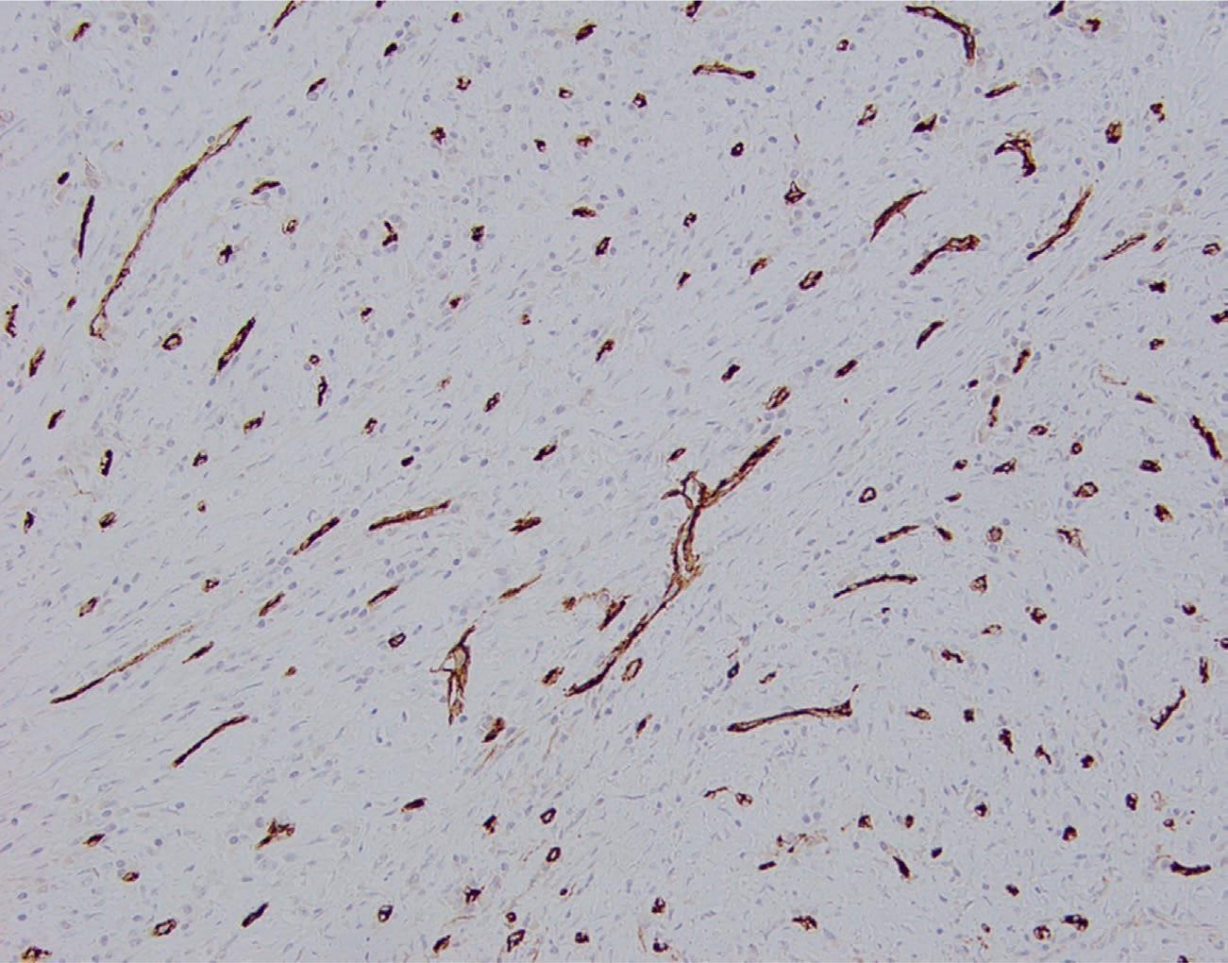
Immunohistochemical staining for CD34 highlights numerous thin vessels in the lesional area (200x magnification via a microscope digital camera).

**Figure 11:**
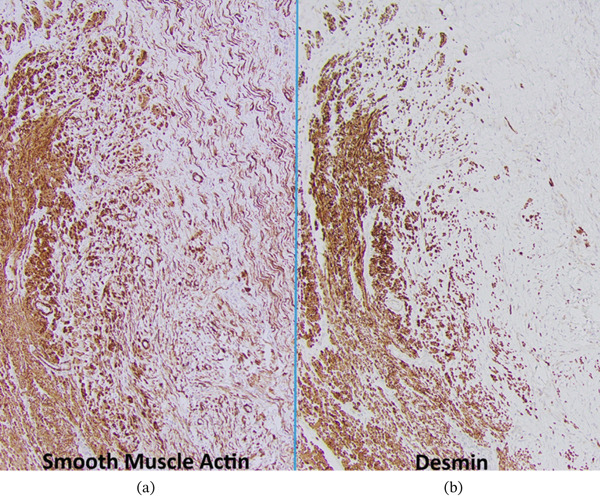
Immunohistochemical staining for smooth muscle actin and desmin. (a) Smooth muscle actin highlights the muscularis propria (b) as compared to lesional cells. (a) Desmin also highlights the muscularis propria and (b) shows no significant expression in lesional cells (100x magnification via a microscope digital camera).

**Figure 12:**
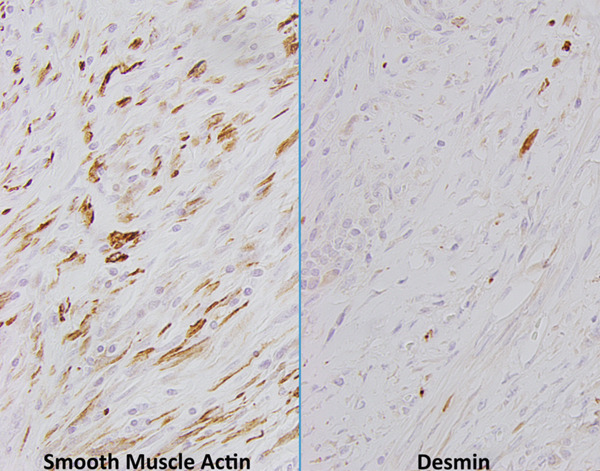
Immunohistochemical staining for smooth muscle actin and desmin. Smooth muscle actin shows variable cytoplasmic expression in the majority of lesional cells. Desmin shows cytoplasmic expression in a subset of lesional cells (400x magnification via a microscope digital camera).

Given the degree of inflammation and unusual nature of the tumor, an immunohistochemical (IHC) workup was decided for further evaluation of the lesion. DOG‐1 (K9‐Leica), CD117 (YR145‐Cellmarque), CD34 (Q BEND10‐Roche), SMA (1A4‐Roche), beta‐catenin (14‐Roche), and desmin (DER11‐Roche) were each performed on two separate blocks of the lesion. The lesional spindle cells did not show significant expression of DOG‐1, CD117, HHV‐8 (13B10‐Cellmarque), or ALK‐1 (ALK‐1‐Agilent). No aberrant nuclear reactivity for beta‐catenin was detected, and CD34 was also nonreactive in the spindle cells. CD34 did highlight abundant small, thin vessels in the lesional area that were not apparent on routine hematoxylin and eosin (H&E) staining (Figure 10). Desmin and SMA stained the muscularis propria as well as a subset of myofibroblastic cells within the tumor (Figures 11 and 12). CD3 (26v6‐Roche) and CD20 (L26‐Agilent) highlighted intermingled lymphocytes, while plasma cells stained for CD138 (B‐A38‐Roche). In the latter population, kappa/lambda in situ hybridization revealed a polyclonal population. Approximately 56% of the IgG+ plasma cells were IgG4+ by immunohistochemistry performed on one of the tissue blocks, with as many as 230 IgG4+ plasma cells identified in a high‐power (400x) field. Serum immunoglobulin G subclass 4 testing was subsequently performed, which was within the normal reference range (result of 83 mg/dL with a reference range of 2–96 mg/dL). The patient had a follow‐up appointment a year later and reported he had been asymptomatic and in good health since his operation.

## 3. Discussion

RNFP involving the abdomen was first described in a small case series from Yantiss et al. published in 2003 [1]. In that article, five cases were described of a mass‐like lesion (sometimes multiple), with subsequent microscopic evaluation revealing a benign reactive process. These cases were described as being proliferations of spindle cells in a collagenous matrix arranged in small, disordered fascicles. Mitotic figures were reported to be uncommon in those cases.

Since that initial publication, a small number of additional cases have been reported, totaling 25 (Table 1). These had a wide range of ages encompassing both pediatric and adult patients (13–72) and a male predominance (64%). The majority of cases were associated with preceding trauma, surgery, or inflammatory process in the area, with only four cases specifically mentioning a lack of this feature. GIST was the most common initial clinical diagnosis of these lesions, although many reports did not specifically mention a differential diagnosis prior to surgery. These tumors do not appear to be restricted to a specific abdominal/pelvic location, with reports spanning the stomach, small intestine, large intestine, pancreas, uterus, and omentum. Several patients had multiple lesions spread throughout the abdominal and pelvic cavities [1, 2, 8–11]. However, more than half (14/25) presented as solitary lesions. These tumors are reported to have a wide range of sizes, ranging from 0.3 to 19.5 cm, and may arise in mesenteric fat, muscularis propria, and/or submucosa.

**Table 1:** Clinical Features in RNFP cases reported to date.

Article	Publication year	Patient age	Patient gender	Clinical follow‐up
Yantiss et al.	2003	48	Male	Follow‐up: At least 10 months for all patients, no recurrence noted
		50	Female	
		53	Male	
		57	Male	
		71	Male	
Zardawi et al.	2004	72	Female	Follow‐up: Unknown
Daum et al.	2004	59	Male	Follow‐up: At least 5 years for four of the eight patients (for whom the patients who were followed were not specified), no recurrence noted
		46	Male	
		1	Male	
		68	Male	
		30	Female	
		65	Female	
		22	Male	
		41	Male	
Saglam et al.	2005	28	Female	Follow‐up: Unknown
Gauchotte et al.	2009	60	Male	Follow‐up: 4 months, no recurrence noted
McAteer et al.	2012	13	Female	Follow‐up: Unknown
Yin et al.	2012	65	Male	Follow‐up: Unknown
Virgilio et al.	2012	71	Male	Follow‐up: 4 years, no recurrence noted
Salihi et al.	2014	45	Female	Follow‐up: Unspecified period, no recurrence noted
Xiao‐Jiang et al.	2014	16	Female	Follow‐up: 2 years, no recurrence noted
Yan et al.	2014	60	Female	Follow‐up: 1 year, no recurrence noted
Ciftci et al.	2015	71	Male	Follow‐up: 8 months, no recurrence noted
Moodley et al.	2018	65	Female	Follow‐up: Unknown
Girsowicz et al.	2018	17	Male	Follow‐up: 5 years, no recurrence noted

Histological features across these cases were similar. The lesions varied from pauci to moderately cellular. All cases had an associated fibrotic/collagenous matrix, which was sometimes hyalinizing. The cells were spindled to stellate in shape, and no significant mitotic activity was noted. Calcifications were reported in six of the cases. Therefore, RNFP could be included in the radiographic differential diagnoses for abdominopelvic lesions with calcifications, along with other gastric tumors that have been reported to contain calcifications, such as gastric adenocarcinoma (particularly the mucinous variant), GISTs, and calcifying fibrous tumor (CFT) [12–14].

Immunohistochemically, all tested cases were positive for vimentin (18/18, Table 1), with most also expressing SMA (22/24) and cytokeratin AE1/AE3 (9/17). Only one case was reported to demonstrate expression of CD34 in the lesional spindle cells (1/21). While the majority of the cases reported in 2003 by Yantiss et al. showed CD117 expression, only one of the subsequently reported cases showed this staining pattern (Table 2) [1, 15]. The lesion we present was also negative for CD117. This is an interesting finding, as it appears CD117 may actually be uncommon in these lesions, but more examples of this entity are needed before drawing definitive conclusions. The presence of keratin expression in over half of these lesions is also intriguing; this finding could make the distinction between this entity and sarcomatoid carcinoma more challenging, particularly on biopsy specimens.

**Table 2:** Immunohistochemical staining patterns in RNFP cases reported to date.

Article	Reported cases	Vimentin	CD117	Desmin	Smooth muscle actin	Muscle‐specific actin	CD34	AE1/AE3	S100	DOG1
Yantiss et al.	5	5 of 5 cases	4 of 5 cases	3 of 5 cases	3 of 5 cases	4 of 5 cases	0 of 5 cases	0 of 5 cases	0 of 5 cases	
Zardawi et al.	1	1 of 1 case	0 of 1 case	0 of 1 case	1 of 1 case		0 of 1 case	0 of 1 case	0 of 1 case	
Daum et al.	8	7 of 7 cases	0 of 8 cases	0 of 8 cases	8 of 8 cases	5 of 7 cases	0 of 7 cases	6 of 7 cases	0 of 8 cases	
Saglam et al.	1	1 of 1 case		0 of 1 case	1 of 1 case		1 of 1 case		0 of 1 case	
Gauchotte et al.	1	1 of 1 case	0 of 1 case		1 of 1 case		0 of 1 case	1 of 1 case	0 of 1 case	
McAteer et al.	1		0 of 1 case		1 of 1 case	1 of 1 case	0 of 1 case	1 of 1 case		
Yin et al.	1									
Virgilio et al.	1	1 of 1 case		0 of 1 case	1 of 1 case		0 of 1 case		0 of 1 case	
Salihi et al.	1									
Xiao‐Jiang et al.	1		0 of 1 case	0 of 1 case	1 of 1 case		1 of 1 case		0 of 1 case	0 of 1 case
Yan et al.	1	1 of 1 case	1 of 1 case		1 of 1 case		0 of 1 case	1 of 1 case	0 of 1 case	0 of 1 case
Ciftci et al.	1	1 of 1 case	0 of 1 case	0 of 1 case	1 of 1 case		0 of 1 case	0 of 1 case		
Moodley et al.	1		0 of 1 case	1 of 1 case	1 of 1 case		0 of 1 case		0 of 1 case	0 of 1 case
Girsowicz et al.	1									

While the similarly appearing CFT has been reported to have a potential association with IgG4‐related disease, RNFP in the abdominopelvic region has not been demonstrated to have any relationship in the current literature [16]. To the knowledge of the authors, only two cases of abdominal RNFP (both resection specimens) have been studied for the presence of IgG/IgG4 IHC expression [10, 15]. The case presented here did demonstrate markedly increased IgG4 plasma cells (> 200/high power field), an elevated IgG4‐to‐IgG ratio (56%), and focally dense lymphoplasmacytic infiltrates; however, this case lacked other histologic features of IgG4‐related disease, such as obliterative phlebitis. A subsequently performed serum IgG4 was found to be in the normal range. Interestingly, an association between similar lesions in the scrotal region (fibrous pseudotumors) and IgG4‐related disease has also been reported [17–20]. It would be highly recommended to perform IgG and IgG4 in future cases of RNFP in the abdominopelvic region to evaluate if this unique lesion could possibly represent a manifestation of IgG4‐related disease. If so, this would add to the variety of initial presentations that can be seen in this disorder and could potentially be included in clinical diagnostic algorithms.

Fibrous pseudotumors involving the testes and scrotum have been reported as early as 1904 [21]. These lesions can also be associated with trauma but often do not have a readily identifiable cause, as compared to the majority of abdominopelvic RNFPs, which typically have some associated trauma or inflammatory condition [21, 22]. These testicular lesions can be positive for SMA, like abdominopelvic RNFP, and can have calcifications [18, 23–25].

**Figure 13:**
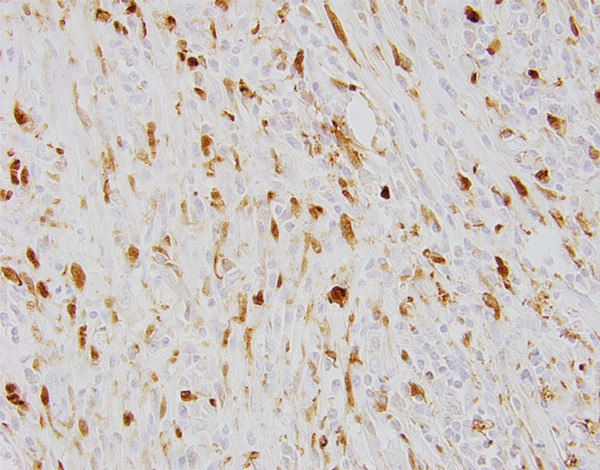
Immunohistochemical staining with Factor 13A showed positive cytoplasmic staining in many of the lesional cells (400x magnification via a microscope digital camera).

It is probable that the most difficult entity to differentiate from nodular fibrous pseudotumor on standard microscopic histologic examination would be CFT. CFT is also composed of fibroblasts but seems to lack myofibroblastic differentiation, as it is typically negative for smooth muscle markers. CFT is also thought to be more cellular than RNFP, but there are many subsequent examples of CFT that are paucicellular [1, 16, 26, 27]. Further complicating matters, a percentage of CFT will stain for smooth muscle markers (including actin) [28, 29]. CD34 has been proposed as a helpful marker to differentiate RNFP from CFT (positive in CFP), but it is unclear what to do in cases where tumors express both CD34 and smooth muscular markers or neither [9, 26, 30–35]. Additionally, there is a significant percentage of cases diagnosed as CFT that do not have any CD34 expression [29]. One marker that may be potentially helpful is Factor XIIIa, as it appears to be expressed in the majority of CFT [29]. However, to our knowledge, other cases of abdominal/pelvic RNFP have not been stained for Factor XIIIa. We performed this stain internally (E980.1‐Biogenex), which did show cytoplasmic expression in many of the lesional stromal cells (Figure 13). However, expression of Factor XIIIa in this entity merits further investigation with a larger cohort. The similar microscopic features and immunophenotypic overlap suggest that these lesions may belong to a spectrum of the same disease process.

There are numerous other spindle cell lesions that can arise in or adjacent to the stomach, including GISTs, inflammatory fibroid polyps (IFPs), inflammatory myofibroblastic tumors (IMTs), mesenteric fibromatosis (MF), schwannomas, and plexiform fibromyxoma (PF). Fortunately, distinguishing these lesions from RNFP is typically possible with careful attention to histologic features, IHC staining patterns, and even molecular testing in some instances. GISTs will have strong and diffuse IHC expression with DOG‐1, while RNFPs are typically negative [36]. IFPs typically contain abundant eosinophils and have characteristic circling of tumor cells around vessels (“onion skinning”) [37]. IFP will also show CD34 IHC expression, unlike the vast majority of RNFP so far reported, and a platelet‐derived growth factor receptor‐*α* (PDGFRA) mutation can be detected by molecular analysis [38]. IMTs can be difficult to distinguish from RNFP on histologic grounds. However, they generally have a more frequent recurrence rate and can also harbor anaplastic lymphoma kinase (ALK) or ROS1 mutations [39]. MF is typically located in the perigastric soft tissues as opposed to the gastric wall and also has a tendency for recurrence [40]. MF also has characteristic aberrant nuclear *β*‐catenin labeling, which has not been reported in RNFP [41].

In summary, RNFP is an intriguing lesion, and references thus far have been limited to case reports and small case series. Of the 25 tumors in the literature reported thus far, the majority express vimentin, SMA, and muscle‐specific actin when those stains were performed. Some aspects of this lesion still need to be explored, including developing a distinct IHC profile to distinguish it from similar lesions, as well as to ascertain if there is any association between this lesion and IgG4‐related disease. Our current case had several notable features, including an acute presentation with severe clinical symptoms, intralesional calcifications that were noted on CT imaging, as well as microscopic calcifications on histologic examination, abundant IgG4‐positive plasma cells, and a lack of IHC expression of CD117.

## Funding

No funding was received for this manuscript.

## Conflicts of Interest

The authors declare no conflicts of interest.
